# Oncolytic virus VG161 offers new hope for refractory liver cancer

**DOI:** 10.3389/fonc.2025.1605177

**Published:** 2025-07-11

**Authors:** Jinqiu Li, Haiyan Zhou, Xinping Wang, Kai Huang, Yanjun Wang

**Affiliations:** ^1^ Infectious Diseases Department, Weifang People’s Hospital, Weifang, Shandong, China; ^2^ Department of Medical Oncology, Qilu Hospital of Shandong University, Jinan, Shandong, China

**Keywords:** hepatocellular carcinoma, oncolytic virus, VG161, cancer immunotherapy, tumor microenvironment

## Introduction

A recent study in Nature introduces VG161, a multi-armed oncolytic herpes simplex virus, as a novel therapeutic strategy for hepatocellular carcinoma (HCC) that has progressed beyond standard treatments (1). In a first-in-human phase 1 trial, Shen et al. demonstrate that VG161 exhibits a favorable safety profile, induces significant tumor responses, and reshapes the tumor immune microenvironment in patients with advanced, treatment-refractory HCC.

## The potential of oncolytic virotherapy in HCC

HCC remains one of the most aggressive and difficult-to-treat solid tumors, accounting for over 800,000 deaths annually worldwide (2). Most HCC cases are diagnosed at advanced stages, particularly in regions with high hepatitis B virus (HBV) prevalence (3). Historically, first-line treatment relied on multikinase inhibitors such as sorafenib and lenvatinib, which offered limited response rates and modest survival benefits.

In recent years, immunotherapy has reshaped the therapeutic landscape. Immune checkpoint inhibitors targeting PD-1 or PD-L1, such as nivolumab and pembrolizumab, showed initial promise, particularly in second-line settings, but overall response remained suboptimal due to the immunosuppressive tumor microenvironment typical of HCC. The approval of atezolizumab combined with bevacizumab has since become a new first-line standard, demonstrating significant improvements in overall and progression-free survival over sorafenib. Other strategies, including dual checkpoint blockade (e.g., durvalumab and tremelimumab) and emerging cellular therapies, are being actively pursued. However, many patients still experience disease progression or are ineligible for immunotherapy due to underlying liver dysfunction or autoimmune contraindications. These limitations underscore the need for novel agents that can both remodel the immune environment and provoke systemic anti-tumor responses. VG161, with its multi-armed design and localized delivery, represents a distinctive immuno-oncology platform that may address these gaps (4).

Oncolytic virotherapy is an approach that has gained traction in recent years. Oncolytic viruses (OVs) are engineered to preferentially infect and destroy tumor cells while sparing normal tissue (5). Their therapeutic benefits extend beyond direct oncolysis; by releasing tumor-associated antigens and danger-associated molecular patterns, they can transform immunologically “cold” tumors into “hot” immune-infiltrated lesions (6). This multifunctionality makes them appealing candidate therapies for cancers like HCC, where immune exclusion poses a significant barrier to effective treatment (7). Earlier efforts using oncolytic viruses, such as JX-594 (pexastimogene devacirepvec), established safety and proof of concept in liver cancer but were ultimately limited by modest clinical efficacy, underscoring the need for more potent and immunologically active constructs (8).

## VG161: a multi-armed oncolytic virus

VG161 exemplifies this next generation of oncolytic virotherapy (9). Built on an HSV-1 backbone, VG161 has been genetically engineered to encode four immunostimulatory molecules: interleukin-12 (IL-12), interleukin-15 (IL-15), IL-15 receptor alpha (IL-15Rα), and a PD-1–PD-L1-blocking fusion protein (10). These components serve distinct yet complementary roles in the anti-tumor immune response. IL-12 promotes Th1 polarization and enhances cytotoxic T lymphocyte (CTL) activity. IL-15 supports memory T cell and NK cell proliferation, while IL-15Rα extends IL-15 half-life and enhances its biological effects. The PD-1–PD-L1-blocking fusion protein inhibits immune checkpoints locally within the tumor, mitigating T cell exhaustion.

To improve tumor selectivity and reduce toxicity, the viral genome includes a deletion of ICP34.5, a neurovirulence gene, which enhances safety. The overall design aims to transform the tumor into an immune-reactive environment while preserving the core mechanism of oncolytic lysis. Compared to JX-594, VG161 incorporates multiple immunostimulatory transgenes—including cytokines and a checkpoint inhibitor—and is designed to more effectively remodel the tumor microenvironment while maintaining tumor-selective oncolysis (**Table 1**). The clinical relevance of this multi-armed construct was investigated in a first-in-human phase 1 trial, summarized below.

**Table 1:** Key features of VG161 and JX-594.

Feature	VG161 (HSV-1)	JX-594 (Pexa-Vec, Vaccinia)
Viral backbone	Herpes simplex virus type 1 (HSV-1)	Vaccinia virus
Key transgenes	IL-12, IL-15, IL-15Rα, PD-1/PD-L1 blocker	GM-CSF
Tumor selectivity	ICP34.5 deletion	Thymidine kinase (TK) inactivation
Delivery route	Intratumoral	Intratumoral or intravenous
Immune activation	Cytokine release, checkpoint blockade	GM-CSF-mediated immune priming
Clinical trial outcome	ORR 17.7%, systemic T cell/NK activation (phase 1)	Safe, but limited efficacy in HCC (phase 1/2)

## Clinical and mechanistic insights from the phase 1 trial

The multicenter phase 1 trial (NCT04806464) enrolled 44 patients with advanced primary liver cancer, all of whom had failed at least two prior lines of systemic therapy. Forty patients with HCC were included in the efficacy analysis. VG161 was administered intratumorally across dose-escalation and dose-expansion cohorts. The treatment was generally well-tolerated. The most frequent adverse events included pyrexia and transient cytopenias, such as decreased lymphocyte and platelet counts, while liver function remained stable and no immune-related adverse events were seen during follow-up. No dose-limiting toxicities occurred, and the maximum tolerated dose was not reached.

Clinically, VG161 demonstrated an objective response rate (ORR) of 17.7% and a disease control rate (DCR) of 64.7% in this heavily pretreated population. Multiple patients showed significant tumor necrosis, and one case notably transitioned from unresectable to resectable disease. Interestingly, non-injected lesions often exhibited more pronounced regression than the directly treated sites, suggesting a systemic immunologic mechanism reminiscent of the abscopal effect.

**Figure 1:**
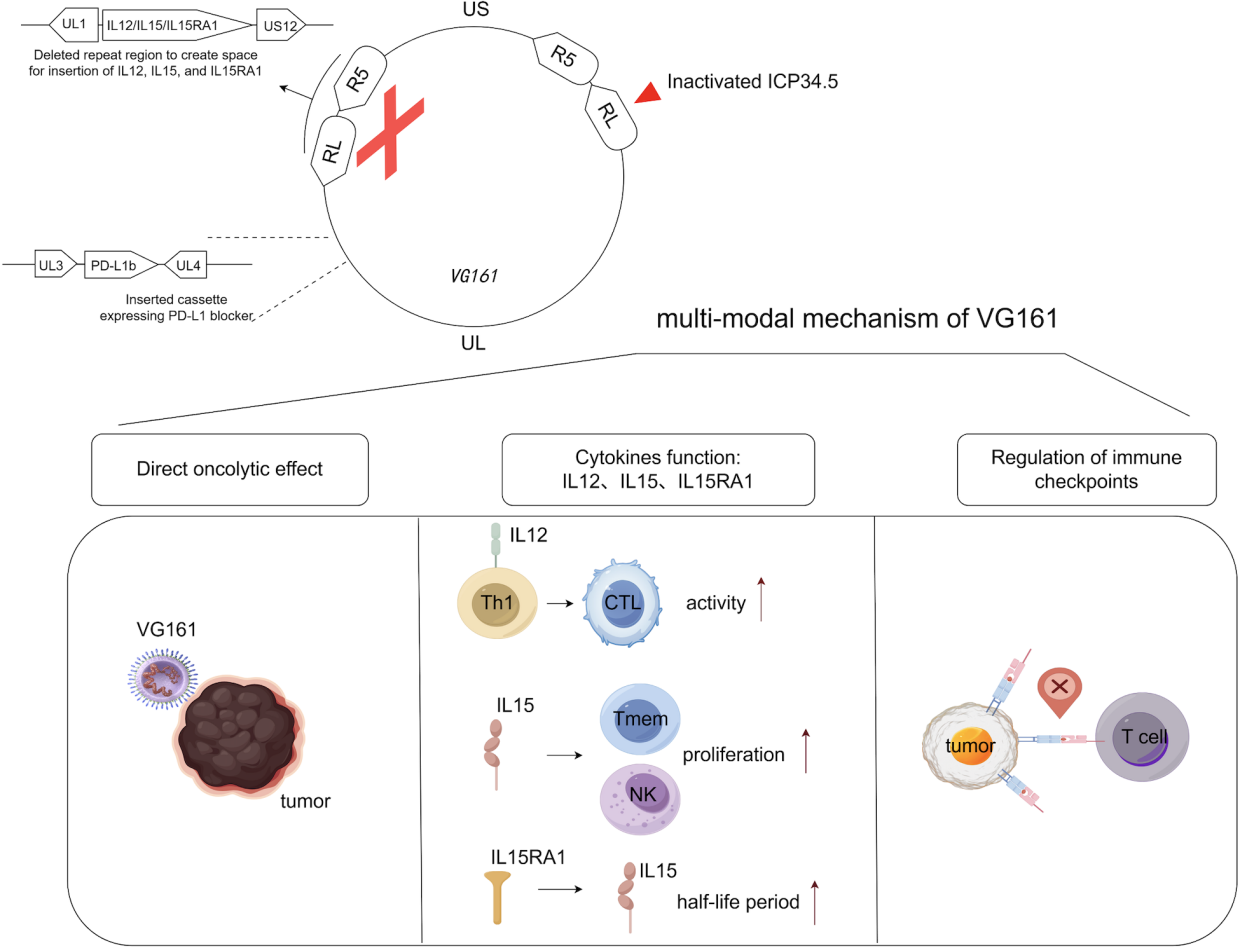
Genomic map of VG161 and the roles of its various components play in anti-tumor immune responses. VG161 was constructed by modifying expanded sections of the prototypic HSV-1 genome, whose UL region is flanked by inverted repeats RL and US region by inverted repeats RS. IL-12 drives Th1 polarization and boosts CTL activity; IL-15 expands memory T/NK cells, with IL-15Rα stabilizing IL-15 and amplifying its effects. Concurrently, a PD-1–PD-L1-blocking fusion protein locally inhibits tumor immune checkpoints to reduce T cell exhaustion. Generated with BioRender.

Mechanistic exploration using single-cell RNA sequencing (scRNA-seq), spatial transcriptomics, and T cell receptor sequencing (scTCR-seq) revealed significant remodeling of the tumor immune landscape. Post-treatment samples exhibited increased infiltration of CD8+ T cells and NK cells, along with expansion of T cell clonotypes. Spatial transcriptomics identified enhanced cell-cell communication in non-injected lesions, particularly among immune populations, suggesting that VG161 can induce a systemic immune response from localized administration (**Figure 1**).

Multiplex immunofluorescence corroborated these findings, demonstrating increased CD3+ and CD8+ T cell presence after VG161 treatment. Notably, the immunologic transformation was more evident in non-injected lesions, raising the possibility that innate antiviral responses triggered by local viral replication—such as type I interferon signaling—may transiently suppress immune cell infiltration or function at the injection site, due to early-phase antiviral immunity, despite the virus’s overall immunostimulatory effects. Nevertheless, the overall pattern was consistent with broad immune activation and a shift from a “cold” to a “hot” tumor phenotype.

## Precision and future utility

In parallel with assessing clinical safety and efficacy, the investigators also sought to identify potential predictive biomarkers to guide patient selection. Recognizing the importance of personalized approaches in immunotherapy, the investigators developed a transcriptomic biomarker model, ViroPredict 1.0, to identify patients most likely to benefit from VG161. Using baseline tumor RNA-seq data, they performed least absolute shrinkage and selection operator (LASSO) Cox regression and identified five genes—SEZ6, AKR1C1, LILRA5, NCKAP5, and SETD9—associated with therapeutic outcomes. The resulting risk score stratified patients into high- and low-benefit groups, with significant differences in progression-free and overall survival.

This model was further validated in The Cancer Genome Atlas (TCGA) liver cancer dataset, where it failed to predict survival in patients not treated with VG161, confirming its specificity for oncolytic virotherapy. This approach marks a significant advance toward precision oncolytic therapy and could eventually guide selection of therapy in clinical settings.

VG161 also demonstrated compatibility with concurrent antiviral treatment. Given the high prevalence of HBV in the HCC population, it is clinically important that antiviral agents like entecavir did not interfere with VG161 replication or therapeutic efficacy. This confirms the feasibility of using VG161 in HBV-positive patients without requiring modification of existing antiviral regimens.

## Discussion

The results of this phase 1 trial place VG161 at the forefront of innovation in the treatment of advanced HCC. By combining direct tumor lysis with potent immune modulation, VG161 addresses key challenges that have limited the success of immunotherapy in this malignancy. Its ability to induce systemic immune responses, even from localized administration, opens the door to broader clinical utility and combination strategies.

One of the most compelling findings was the apparent immunological shift in the tumor microenvironment after VG161 treatment. Through scRNA-seq and spatial transcriptomics, the study demonstrated increased immune infiltration, heightened TCR clonality, and enhanced immune signaling. These findings support the hypothesis that VG161 not only disrupts tumor cells but also acts as an *in situ* immunological “re-education” agent.

Challenges remain in optimizing delivery, especially for deep-seated or multifocal tumors that are difficult to access via intratumoral injection. Future development may involve engineering systemic delivery vectors or using cell-based carriers to enhance tumor targeting. For example, mesenchymal stem cells and T cells have been investigated as delivery vehicles for OVs due to their tumor-homing capacity. Encapsulation strategies using liposomes or polymeric nanoparticles may also help shield viruses from neutralizing antibodies in circulation and improve systemic biodistribution. In addition, the phase 1 trial’s small sample size, lack of a control group, and preliminary nature of the biomarker model should be acknowledged when interpreting the results. Additionally, the integration of VG161 into earlier lines of treatment, such as in the neoadjuvant setting or in combination with checkpoint blockade, could further expand its clinical impact. Combination strategies may be particularly useful in enhancing systemic immune responses, especially in immune-excluded tumors.

The development of ViroPredict 1.0 provides a foundation for tailoring treatment to those most likely to benefit, addressing a major hurdle in the clinical adoption of OVs. Its validation in future trials will be critical, and further refinement may include integration with spatial or single-cell signatures.

This study also highlights an important aspect of global health equity. Given that a large proportion of HCC cases worldwide are HBV-related, a therapy like VG161 that retains efficacy in HBV-positive patients and is compatible with standard antiviral treatment holds significant potential for widespread clinical application, particularly in Asia and sub-Saharan Africa. However, the cost and accessibility of transcriptomic biomarkers such as ViroPredict 1.0 may present challenges in low-resource settings, and ethical considerations regarding biomarker-based patient selection should be carefully considered.

In conclusion, VG161 represents a new generation of oncolytic immunotherapy that integrates tumor-selective viral lysis with targeted immune activation. Rather than functioning solely as a cytolytic agent, VG161 is designed as a programmable immune modulator—capable of reshaping the tumor microenvironment, eliciting systemic immune responses, and enabling patient stratification through predictive biomarkers. These combined features suggest that VG161 is not just a therapeutic candidate, but a platform with the potential to redefine the clinical application of oncolytic virotherapy in solid tumors.
